# Searching for a sign of exotic *Aedes albopictus* (Culicidae) introduction in major international seaports on Kyushu Island, Japan

**DOI:** 10.1371/journal.pntd.0009827

**Published:** 2021-10-06

**Authors:** Chao Yang, Toshihiko Sunahara, Jinping Hu, Kyoko Futami, Hitoshi Kawada, Noboru Minakawa

**Affiliations:** 1 Department of Vector Ecology and Environment, Institute of Tropical Medicine, Nagasaki University, Nagasaki, Nagasaki, Japan; 2 Program for Nurturing Global Leader in Tropical and Emerging Communicable Diseases, Graduate School of Biomedical Sciences, Nagasaki University, Nagasaki, Nagasaki, Japan; The University of Kansas, UNITED STATES

## Abstract

**Background:**

The Asian tiger mosquito, *Aedes albopictus*, has spread around the world. The migration was mainly mediated by maritime transportations. This species is known as an efficient vector for arboviruses, and it was responsible for the recent dengue outbreak in Tokyo, Japan. As the vector competence varies among geographical populations, and insecticide resistant populations have emerged, it is important to reveal their movements. The present study uses molecular techniques to search for a sign of introduction of an exotic population in three major international seaports on Kyushu Island.

**Methodology/principal findings:**

Adults of *Ae*. *albopictus* were sampled around the international seaports of Fukuoka, Kitakyushu, and Nagasaki. Pairwise fixation indexes were estimated between the sampled populations based on 13 microsatellite markers. There was no clear genetic differentiation between distant and port populations in Kitakyushu and Nagasaki. However, the analysis found one distinct group near the container terminal in Fukuoka, which handles international freight containers mainly from adjacent countries. DNA samples were also obtained from Goto, Tsushima, Honshu, Ryukyu, Thailand, and the Philippines; and a cluster analysis and discriminant analysis revealed that the distinct group in Fukuoka did not belong to these groups. Combined with the results of phylogenetic analysis based on CO1, these results implied that this group originated from one Asian temperate region outside of Japan. Neutrality test and mismatch distribution analysis suggested that the establishment of this group was not recent.

**Conclusions/significance:**

The present study found a sign of *Ae*. *albopictus* introduction from a temperate region of Asia through maritime freight container transportation. The genetically distinct group found in Fukuoka likely originated from a temperate region outside of Japan. Maritime container transportation may introduce to Japan mosquitoes with greater vector competence/insecticide resistance. This is the first study to describe the spatial population structure of *Ae*. *albopictus* in Japan using molecular techniques.

## Author summary

*Aedes albopictus* is an invasive species and an efficient disease vector. The bioecological traits of this species and human-mediated transportations have fueled its worldwide spread. Because the levels of vector competence and insecticide resistance vary among geographical strains, the worldwide migration of this species is a concern for public health and vector control. While numerous studies have been done in non-native areas, within its native areas in Asia movements have not been explored. This study used molecular techniques to search for signs of introduction of an exotic population through three major international seaports on Kyushu Island, Japan. The present study found a genetically distinct group of *Ae*. *albopictus* near the container terminal in Fukuoka. The study suggests that its origin was one temperate region of East Asia outside of Japan, and the establishment of this group was not recent. As vector competence varies among different populations and the knock down resistance gene has been reported from some populations outside Japan, the risk of introducing mosquitoes with greater vector competence/insecticide resistance needs to be considered. This is the first study to describe the spatial population structure of *Ae*. *albopictus* in Japan using molecular techniques.

## Introduction

The Asian tiger mosquito, *Aedes albopictus* (Skuse, 1894), is indigenous to East Asia [1,2]. Because of its diapause eggs and successful adaptation to the human environment [1–5], this species has rapidly spread around the world with an increase of economic activities [6,7]. The migration was mainly mediated by maritime transportation [6]. For instance, the species was imported to the USA through the used tire trade with container ships [6]. The mosquito was further introduced to neighboring countries from the USA by subsequent transport of some of the imported tires [6]. The Global Invasive Species Database has listed this species as one of 100 worst invasive species [8].

*Aedes albopictus* is an efficient vector for arboviruses [1,2], and is responsible for outbreaks of dengue and zika in several invaded areas [9–12]. In Italy this species was responsible for Chikungunya outbreaks [13,14], and reportedly has a greater susceptibility to Chikungunya virus compared with its sister species, *Aedes aegypti* [15]. Despite the presence of *Ae*. *albopictus*, Japan is not endemic for the arboviral diseases transmitted by this mosquito. However, occasional autochthonous transmissions of dengue viruses (DENVs) occurred in 1942–1944 [16], and most recently in 2014 and 2019 [17,18]. Although the DENVs might have been introduced by humans, mosquito-mediated introduction is still possible [19]. *Aedes albopictus* is able to pass DENVs to progeny transovarially [20], which may diversify the routes of virus introduction.

As the vector competence of *Ae*. *albopictus* varies among different geographical populations [21,22], the possible introduction of individuals with greater vector competence has been a public health concern. It is reported that Japanese *Ae*. *albopictus* had lower competence for dengue virus than populations from Malaysia and Hawaii [21]. *Aedes albopictus* populations in Japan lack the knockdown resistance (*kdr*) mutation in the voltage-gated sodium channel (VGSC) [23]. However, *kdr* has been reported recently from China, Singapore, and other locations [24–29], and thus a potential introduction to Japan raises a public health concern. No migration research of *Ae*. *albopictus* has been conducted, while invasions of *Ae*. *aegypti* in international airports in Japan are occasionally found by larval research and ovitraps [30]. Considering that *Ae*. *aegypti* cannot overwinter in Japan [31], the threat by imported *Ae*. *albopictus* is greater because of its overwintering ability by diapause eggs. Thus, for risk management of arbovirus transmissions in Japan it is important to monitor *Ae*. *albopictus* introduction at international entry points. However, unlike the situation for *Ae*. *aegypti* [31], it is impossible to distinguish introduced *Ae*. *albopictus* morphologically from local populations. To identify introduced individuals from local populations, genetic analysis using molecular markers such as microsatellites is a suitable method. High polymorphism has been observed in some determined microsatellite loci of *Ae*. *albopictus* [32] and previous studies had successfully revealed local population structures in South-east Asian and Oceanic countries [33,34], USA [35], and European countries [36]. In Germany, repeated introduction of this species is suggested [36].

Japan and adjacent countries have a lengthy trading history, with long-term and fixed traffic patterns. Cargo entering Japan by ships greatly exceeds that by airplanes, thus increasing the chance of the importation of exotic *Ae*. *albopictus*. Introduction of foreign mosquitoes would influence the genetic structure of the local Japan populations, and might lead to a transformation of vector competence and insecticide resistance, thus complicating arbovirus transmission and vector control. The present study hypothesized that maritime transportations facilitate the introduction of exotic *Ae*. *albopictus* individuals to Japan. To test this hypothesis, we used microsatellite and cytochrome c oxidase subunit 1 (CO1) molecular techniques to explore the possibility that exotic *Ae*. *albopictus* has entered the major international seaports on Kyushu Island. Specifically, this study determined if the genetic structures of *Ae*. *albopictus* populations in the seaports and their vicinities are distinct from those of other local populations. The study also inferred the origins of the seaport populations by comparing their genetic structures and phylogeographic relationship with those of other Japanese and oversea populations.

## Materials and methods

### Mosquito collection

**Fig 1:**
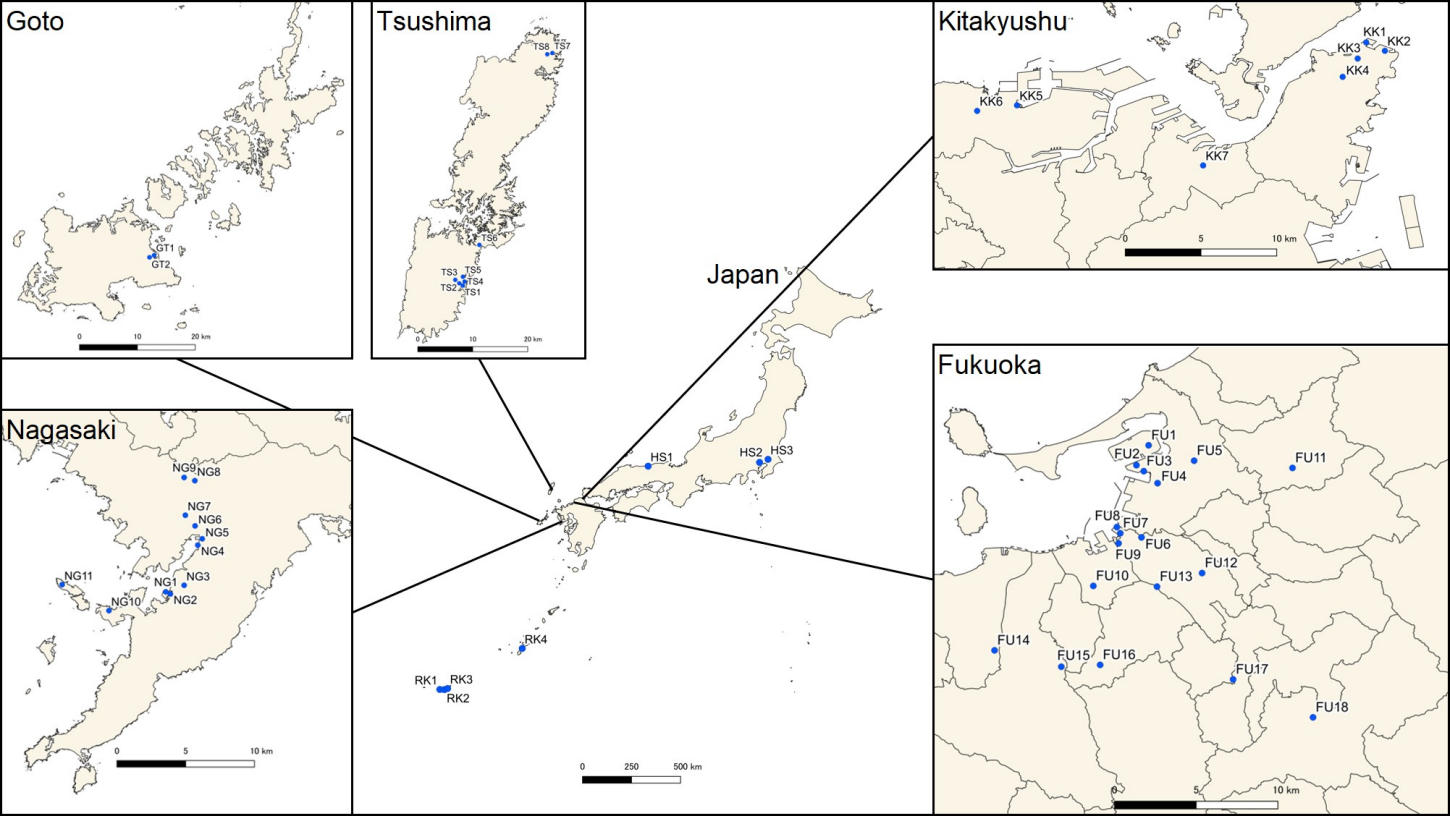
Collection sites of *Ae*. *albopictus* in Kyushu. Created by processing Free vector and raster map data @ naturalearthdata.com and National Land Numerical Information (Administrative Area Data) @ Ministry of Land, Infrastructure, Transport and Tourism, Japan (https://nlftp.mlit.go.jp/ksj/gml/datalist/KsjTmplt-N03-v3_0.html).

To examine signs of introduction of *Ae*. *albopictus* from abroad, we compared the genetic structure of adult mosquitoes collected in three cities on Kyushu Island, Fukuoka, Kitakyushu, and Nagasaki, the locations of the busiest international terminals. Accordingly, it was sought to observe genetic differences between ship terminals and distant areas due to a population introduced via maritime transportation. In each city, three areas were designated for sampling: two areas around the terminals and one distant area. Fukuoka has two large container terminals; Island City Terminal and Kashii Park Terminal. These terminals are located next to each other in the northern part of Hakata Port (Fig 1). The former terminal imported 3.5 million tons and the latter imported 5.7 million tons of international cargo in 2018 (Hakata Port annual report 2018). Approximately 65% of the combined cargo from the terminals was imported from China and South Korea. Adults of *Ae*. *albopictus* were sampled from five vicinity sites of the two terminals using sweep nets in September 2019. A vicinity was considered as an area within 4 km of a terminal. At each collection site, one to three collectors swept nets for a maximum of 20 minutes or until 20–30 adults were obtained, typically around shaded places near bushes for efficient collection. Mosquitoes were also sampled from five vicinity sites of Chuo Terminal approximately 5 km south of the container terminals in September 2018, September 2019, and June 2020. This terminal imported 0.4 million tons of non-container cargo mainly from China and South Korea in 2018. The terminal also handled international passenger ships, mostly from South Korea, and 276 cruise ships in 2018. For further comparison, mosquitoes were sampled in June 2020 from eight inland sites distanced at least 6 km from each terminal (Fig 1 and Table 1). These sites were selected based on accessibility and available sampling time.

**Table 1:** Sampling information of each *Ae*. *albopictus* population.

Origin	Population	Code	Category	Area	Coordinate	Collection year	N
Fukuoka	Island city Terminal	FU1	Port	Container terminal area	33.6543401N,130.4129074E	2019	12
	Kashii Park Terminal	FU2	Port		33.6536714N,130.4123493E	2019	5
	Minatohyakunen-park	FU3	Vicinity		33.6488956N,130.4172075E	2019	11
	Matsusakichuo-park	FU4	Vicinity		33.6410158N,130.4262649E	2019	25
	Miyanodaichuo-park	FU5	Vicinity		33.6557937N,130.4503261E	2019	19
	Higashi-park	FU6	Vicinity	Chuo terminal area	33.6053309N,130.4156933E	2019	21
	Chuo-Terminal_2019	FU7	Port		33.6084893N,130.4009176E	2019	14
	Chuo-Terminal_2018	FU8	Port		33.6108720N,130.3994932E	2018	11
	Sunset-park	FU9	Vicinity		33.6013867N,130.4005986E	2019	22
	Minami-park	FU10	VIcinity		33.5733738N,130.3839956E	2020	17
	Hisayama-park	FU11	Inland	Distant area	33.6510411N,130.5150458E	2020	14
	Higashihirao-park	FU12	Inland		33.5818358N,130.4555478E	2020	27
	Heiseinomori-park	FU13	Inland		33.5728789N,130.4259146E	2020	30
	Kanatakenosato-park	FU14	Inland		33.5305383N,130.3206488E	2020	13
	Nishiaburayama-park	FU15	Inland		33.5305383N,130.3206488E	2020	9
	Kashibara-park	FU16	Inland		33.5213808N,130.3884592E	2020	8
	Hinouraike-park	FU17	Inland		33.5118255N,130.4759832E	2020	4
	Ishizaki-town	FU18	Inland		33.4868488N,130.5285499E	2020	27
Kitakyushu	Tachiura-Terminal_T1	KK1	Port	Tachiura terminal area	33.9661191N,131.0015498E	2019	25
	Tachiura-Terminal_T2	KK2	Port		33.9609639N,131.0144422E	2019	34
	Seizan-park	KK3	Vicinity		33.9575055N,130.9933783E	2019	11
	Narutake-park	KK4	Vicinity		33.9419628N,130.9628058E	2019	15
	Hibiki Terminal	KK5	Port	Hibiki terminal area	33.9187488N,130.7245466E	2019	23
	Greenland-park	KK6	Vicinity		33.9180470N,130.7238950E	2019	13
	Nakatsugachi-park	KK7	Middle	Distant area	33.8791480N,130.8849100E	2019	25
Nagasaki	Yanagi Terminal	NG1	Port	Yanagi terminal area	32.7047190N,129.8447339E	2019	28
	Kogakura-town	NG2	Vicinity		32.7031010N,129.8482975E	2019	14
	Shintomachi-park	NG3	Vicinity		32.7098199N,129.8589354E	2019	5
	Matsugae Terminal	NG4	Port	Matsugae terminal area	32.7388338N,129.8662138E	2019	22
	Kabashimamachi-park	NG5	Vicinity		32.7450116N,129.8716788E	2019	9
	Tateyama-park	NG6	Vicinity		32.7558276N,129.8673928E	2019	24
	Yanagawa-park	NG7	Vicinity		32.7641050N,129.8598655E	2019	15
	Showa-park	NG8	Inland	Distant area	32.7908756N,129.8672160E	2019	20
	Sumiyoshi-park	NG9	Inland		32.7933462N,129.8589228E	2019	20
	Kouyagi-park	NG10	Coast		32.6844027N,129.8002950E	2019	21
	Ioujima-park	NG11	Coast		32.7102978N,129.7669241E	2019	31
Goto	Fukue Port	GT1	Port	N/A	32.6949537N,128.8483495E	2019	5
	Wakamiya-shrine	GT2	Vicinity	N/A	32.6947093N,128.8465768E	2019	6
Tsushima	Izuhara Port	TS1	Port	N/A	34.1977071N,129.2919288E	2019	29
	Imayashiki-park	TS2	Vicinity	N/A	34.2021556N,129.2898677E	2019	8
	Kinen-park	TS3	Vicinity	N/A	34.2023263N,129.2878448E	2019	17
	Isaribi-park	TS4	Vicinity	N/A	34.2038890N,129.2904493E	2019	14
	Hiyoshidai-park	TS5	Vicinity	N/A	34.2101025N,129.2912448E	2019	11
	Green-park	TS6	Distant	N/A	34.2780598N,129.3245897E	2019	11
	Hitakatsu Port	TS7	Port	N/A	34.6569080N,129.4684646E	2019	18
	Toyosaki-shrine	TS8	Vicinity	N/A	34.6544880N,129.4584783E	2019	6
Ryukyu Islands	Iriomote-jima	RK1	N/A	N/A	24.4351307N,123.7726704E	2017	3
	Kohama-jima	RK2	N/A	N/A	24.3305209N,123.9507397E	2017	14
	Ishigaki-jima	RK3	N/A	N/A	24.4713047N,124.0649114E	2017	18
	Okinawa-jima	RK4	N/A	N/A	26.2516916N,127.7662191E	2019	8
Honshu	Tottori	HS1	N/A	N/A	35.5150146N,134.1565672E	2016	21
	Tokyo	HS2	N/A	N/A	35.7043023N,139.7151643E	2020	20
	Ibaraki	HS3	N/A	N/A	35.8574217N,140.1394149E	2019	23
Philippines	Philippines	PHL	N/A	N/A	N/A	2013	19
Thailand	Thailand	THA	N/A	N/A	10.0060109N,98.6307391E	2019	32
						Total	927

N/A: not applicable

Kitakyushu has three container terminals, the adjacent Tachiura Terminals 1 and 2, and Hibiki Terminal 22 km distant (Fig 1). Hibiki Terminal and the two Tachiura terminals imported 1.3 and 1.8 million tons in 2018, respectively. Nearly 75% of containers were imported from China and South Korea. Adult mosquitoes were sampled in September 2019 from four vicinity sites of the Tachiura terminals and two vicinity sites of Hibiki Terminal. For reference, mosquitoes were sampled from one site at the middle point between the terminals (Fig 1 and Table 1).

Nagasaki has two international terminals, Yanagi Terminal and Matsugae Terminal, which are separated by a distance of over 4 km (Fig 1). Matsugae Terminal hosts cruise ships, and Yanagi Terminal hosts container ships and cruise ships. The latter imported 0.1 million tons of cargo that was mainly from Australia (32%) and South Korea (23%) in 2017. The terminals hosted 220 cruise ships, which in 2018 was the second largest port in Japan after Fukuoka. Adult mosquitoes were sampled in July 2019 from three vicinity sites of Yanagi Terminal and four vicinity sites of Matsugae Terminal. For comparison, mosquitoes were sampled from another two coastal sites and two inland sites distanced at least 6 km from each terminal in July and August of 2019 (Fig 1 and Table 1).

Adult mosquitoes were also sampled from Goto Island and Tsushima Island which are located between Kyushu Island and the Korean Peninsula. As Tsushima Island has sea connectivity with both sides and Goto Island has sea connectivity with Kyushu Island, the genetic information of the island mosquito populations was used for inferring the origin of mosquitoes found in the ports on Kyushu Island. Mosquitoes were sampled in September 2019 from two local ports, five vicinity sites, and one middle site on Tsushima Island; and one port and one vicinity site on Goto Island (Fig 1 and Table 1). For reference sites outside of Kyushu, adult mosquitoes were sampled from Honshu (Tottori, Tokyo, and Ibaraki) and Thailand (Ranong) during 2016 to 2020 (Fig 1 and Table 1). The present study also included genetic samples of adult *Ae*. *albopictus* from the Philippines (unknown locality) collected in 2013 and from Ryukyu Islands (four islands) collected in 2017 and 2019, which deposited in the Institute of Tropical Medicine, Nagasaki University (Fig 1 and Table 1).

Collected mosquitoes were immediately placed in 100% alcohol or silica beads and stored in freezers under -25°C. Before molecular analysis, the samples were identified morphologically using the key by Tanaka et al. [37].

### Microsatellite genotyping and analysis

DNA was extracted from up to three legs of each mosquito sample using REDExtract-N-Amp Tissue PCR Kit (SIGMA, St. Louis, USA). Extracted DNA was genotyped based on 13 microsatellite primers according to Beebe et al. [33]. A 10 μl reaction mixture was made with 1 μl of template DNA, 0.2 μM of FAM-labeled M13 fluorescent primer (SIGMA-ALDRICH, St. Louis, USA), 0.1 μM of M13-tailed forward primer for each locus (S1 Table), 0.2 μM of reverse primer for each locus (S1 Table), 1X concentration of Takara ExTaq Buffer, 200 μM of dNTP, 0.25 U of Takara ExTaq (Takara BIO, Inc. Kusatsu, Japan), and 6.65 μl distilled water. The thermocycling involved an initial denaturation at 95°C for 3 mins, and 35 cycles of 95°C for 30 sec, and primer-specific annealing temperatures ranging from 50°C to 57°C for 40 sec (S1 Table), 72°C elongation for 30 sec, and a final extension at 72°C for 5 mins. Then, 1 μl of the resultant PCR fragments were electrophoresed with 9 μl mixture of GeneScan 500 ROX Size Standard (Applied Biosystems, ABI, Foster, USA) and formamide solution on an ABI3730 DNA Analyzer (Applied Biosystems, ABI, Foster, USA) after heating at 95°C for 5 mins and cooling down with an ice plate. Allele sizes were scored using the GeneMapper Software 5 (S2 Table; Applied Biosystems, ABI, Foster, USA).

Fixation index (F), allelic richness (N_a_), the number of effective alleles (N_e_), and the observed (H_o_) and expected (H_e_; unbiased estimate: uH_e_) values of heterozygosity were calculated using GenAlEx (ver. 6.5; S3 Table) [38,39]. Genetic differences between populations were estimated with pairwise fixation index (F_st_) using Genepop (ver. 4.7) [40,41] with G test at a significance level of 0.05 and derived cluster dendrogram based on F_st_ was conducted using stats package in R (ver. 4.0.2). The difference in genetic variation among three areas in each city was assessed using an analysis of molecular variance (AMOVA) (Arlequin ver. 3.5.2.2) [42]. The analysis was also conducted for each pair of the areas in each city. Cluster analysis and discriminant analysis of principal components (DAPCs) were used to reveal the population structures using STRUCTURE (ver. 2.3.4) and adegenet package (ver. 2.0.1) in R (ver. 4.0.2), respectively [43,44]. The population clusters (K) inferred by the cluster analysis were run from K = 1 to 10 based on a pre-run. Each run was conducted with 200,000 burn-in followed by 1,000,000 sampling, using prior information of collection location and using an allele frequency correlated model for 10 independent runs as replication [45]. The best K value was determined according to Evanno’s criteria [46]. Membership coefficient (Q) of the populations was partitioned according the best K using DISTRUCT on the CLUMPAK server [47,48].

### Cytochrome c oxidase subunit 1 (CO1) sequencing and analysis

Mitochondrial DNA cytochrome c oxidase subunit 1 (CO1) sequences were determined to investigate the genetic structure in Japan and to estimate the origin of a distinct population. To examine the CO1 polymorphisms of samples, DNA was amplified with two set of primers developed by Zhong et al. [49]: 1454F (5’ GGTCAACAAATCATAAAGATATTGG 3’) and 2160R (5’ TAAACTTCTGGATGACCAAAAAATCA 3’); and 2027F (5’ CCCGTATTAGCCGGAGCTAT 3’) and 2886R (5’ ATGGGGAAAGAAGGAGTTCG 3’). A 10 μl reaction mixture that contained 1 μl of extracted DNA template, 1X concentration of Takara ExTaq buffer, 200 μM of dNTP, 0.4 μM of each primer, 0.25 U of Takara ExTaq (Takara BIO, Inc. Kusatsu, Japan), and 6.35 μl of distilled water was prepared for PCR amplification under the following thermal condition: an initial denaturation at 94°C for 3 mins followed by 35 cycles at 94°C for 30 sec, 55°C for 30 sec, and 72°C for 1 min, and a final extension at 72°C for 10 mins. Generated PCR products were visualized by separation on 1% agarose gels, then purified with ExoSAP-IT (Affymetix, Inc. Santa Clara, USA) and dyed using bi-directional primers of each set separately with ABI Big Dye Terminator v1.1 Cycle Sequencing Kits (Applied Biosystems, ABI, Foster, USA), and then sequenced on a 3730 DNA Analyzer (Applied Biosystems, ABI, Foster, USA) after ethanol precipitation. Cytochrome c oxidase subunit 1 (CO1) sequences were aligned and edited manually using MEGA7 [50].

A TCS haplotype network was constructed using PopArt1.7 (Population Analysis with Reticulate Trees) with 1000 iterations [51,52]. Deviations from selective neutrality were tested with Fu’s *Fs* statistics [53] and Tajima’s D [54], which was used to examine recent population expansion/bottleneck. Mismatch analysis was conducted using Arlequin (ver. 3.5.2.2) [42] and DnaSP (ver. 6) [55] under the model of population expansion when the putative introduced population was confirmed. The validity of the estimated demographic model was evaluated by the tests of Harpending’s raggedness index (*Hri*) [56] and the sum of squared differences (SSD) [57].

To detect putative sources of introduced populations, a phylogenetic tree was constructed using Bayesian inference (BI) with the Markov Chain Monte Carlo (MCMC) algorithm on BEAST (ver. 1.10.4) [58]. Ten million iterations of MCMC chains were run with sampling every 1,000 iterations, and the first 10% iterations were discarded. Before constructing a phylogenetic tree, the best-fit substitution model (TN+F+R2 model) was selected based on the Akaike information criterion (AIC), corrected AIC (AICc), and Bayesian information criterion (BIC), using IQ-TREE (ver 1.6.12) [59]. All taxa on the tree were assigned into haplogroups based on the definition by Battaglia et al. [60] to estimate whether the haplotype originated from a temperate or tropical region. The haplotypes generated by the present study and those from Battaglia et al. [60] were used to construct the tree. Additional CO1 sequences on the tree were retrieved at the NCBI nucleotide collection database (2020/12/25) restricted to *Ae*. *albopictus*. Only sequences that were hit with over 95% query coverage were used, by means of LC591859 as a query to conduct a blastn screen. A total of 524 sequences were retrieved. The analysis used only sequences that were found in the indigenous areas and after trimming had at least one identical sequence in other sources. The tree topology was visualized with FigTree (ver. 1.4.4) [61].

## Results

### Comparison within local populations using microsatellite analysis

A total of 927 *Ae*. *albopictus* adults was collected from 55 sites (Table 1). Pairwise F_st_ values were calculated using microsatellite data. In Fukuoka 47% (72/153) of F_st_ values were over 0.05. Low F_st_ values (<0.05) were observed among five populations in the container terminal area (FU1 –FU5) except that between FU2 and FU3 (F_st_ = 0.0644, P < 0.05). F_st_ values among eight populations in the distant area (FU11 –FU18) were also low with 89% (25/28) of the values less than 0.05. Five populations in the Chuo terminal area showed a moderate genetic difference with four of ten F_st_ values more than 0.05. F_st_ values between populations of different areas were relatively high with 61% (64/105) of the values more than 0.05 (Table 2). Populations in the three areas (KK1-4, KK5-6, and KK7) of Kitakyushu showed little genetic differentiation (F_st_<0.05) within and between the areas (Table 3). Similarly, populations in the three areas (NG1-3, NG4-7, and NG8-11) of Nagasaki showed little genetic differentiation (F_st_ < 0.05) within and between different areas (Table 4).

**Table 2:** Pairwise fixation index values (F_st_) between eighteen *Ae*. *albopictus* populations in Fukuoka.

	FU1	FU2	FU3	FU4	FU5	FU6	FU7	FU8	FU9	FU10	FU11	FU12	FU13	FU14	FU15	FU16	FU17
FU2	0.0134																
FU3	-0.0021	**0.0644** *															
FU4	0.0275*	0.0157	0.0279*														
FU5	0.0115*	0.0105	0.0166	0.0082													
FU6	0.0089	0.0153	0.0133	0.0136	0.0132												
FU7	**0.0793** **	**0.1311** **	**0.0903** **	**0.1021** **	**0.1038** **	**0.0706** **											
FU8	0.0552**	**0.0673**	**0.0913** **	**0.0877** **	**0.0558** **	**0.0573** **	0.0265**										
FU9	**0.078** **	**0.0618** **	**0.0995** **	**0.0775** **	**0.0589** **	**0.0583** **	**0.0511** **	0.0179*									
FU10	0.0408**	0.0409	**0.0617** **	0.0484**	0.0438**	0.0394**	0.0334**	0.001	0.0201**								
FU11	**0.0809** **	**0.0705** **	**0.1056** **	**0.079** **	**0.0505** **	**0.0986** **	**0.1238** **	**0.0762** **	**0.0513** **	**0.0509** **							
FU12	0.0423**	0.0449	**0.0768** **	**0.0595** **	0.0396**	**0.0671** **	**0.0545** **	0.0202*	0.0384**	0.0068	0.0242**						
FU13	**0.0686** **	**0.0732** **	**0.1038** **	**0.0693** **	0.0496**	**0.0858** **	**0.0831** **	0.0487**	**0.05** **	0.028**	0.0101	0.0042					
FU14	**0.0866** **	**0.0722**	**0.0932** **	**0.0904** **	**0.0806** **	**0.0962** **	**0.0786** **	**0.0746** **	**0.0738** **	0.0471**	**0.0599** **	0.0275	0.0436**				
FU15	**0.0553** **	0.0105	**0.0751** **	0.0452	0.0394	0.0312	0.0245	0.0239	0.0053*	-0.0032	0.0337*	-0.0025	0.0088	-0.0012			
FU16	**0.0991** **	**0.117** **	**0.1364** **	**0.0954** **	**0.0818** **	**0.09** **	**0.0708** **	**0.0545** **	0.0401**	0.0405**	**0.0575** **	0.024	0.0196	**0.0917** **	0.024		
FU17	**0.0567**	0.036	**0.0718**	**0.0662**	0.0476	**0.063**	0.0363	0.0231	0.0118	-0.0084	0.0048	-0.0345	-0.0094	-0.0129	-0.0418	0.0041*	
FU18	0.0459**	**0.0614** **	**0.0682** **	**0.071** **	0.047**	**0.0651** **	0.0379**	0.0281**	0.0408**	0.019**	0.0378**	0.0058	0.0105	0.0282	-0.0021	0.0312	-0.023

*: P < 0.05

**: P < 0.01. The values of F_st_ > 0.05 were indicated in bold.

**Table 3:** Pairwise fixation index values (F_st_) between seven *Ae*. *albopictus* populations in Kitakyushu.

	KK1	KK2	KK3	KK4	KK5	KK6
KK2	0.0147**					
KK3	0.0044	0.0232**				
KK4	0.0086	0.0263**	0.0032			
KK5	0.0054	0.0221**	0.0167	0.0053*		
KK6	-0.0139	0.0121*	-0.0033	0.0119*	0.0059	
KK7	0.0241**	0.0317**	0.0258*	0.038**	0.0087**	0.044**

*: P < 0.05

**: P < 0.01

**Table 4:** Pairwise fixation index values (F_st_) between eleven *Ae*. *albopictus* populations in Nagasaki.

	NG1	NG2	NG3	NG4	NG5	NG6	NG7	NG8	NG9	NG10
NG2	0.0149*									
NG3	0.0117	-0.0143								
NG4	0.0335**	-0.0024	0.025							
NG5	0.0459**	0.0116	0.023	0.0194*						
NG6	0.0297**	0.0024	0.0144	0.0213**	0.0277*					
NG7	-0.0033	-0.0033	-0.0049	0.0012	0.0278**	-0.0001				
NG8	0.0225**	0.0014	-0.0125	0.0116	0.0229	0.015**	-0.0044			
NG9	0.0381**	0.0125**	-0.0122	0.0173*	0.0155*	0.0269**	0.0124*	-0.0026		
NG10	0.0166*	-0.0084	-0.0005	0.0112*	0.0111	0.0193**	-0.0033	0.0172**	0.0142**	
NG11	0.0378**	0.0231**	0.0283**	0.0274**	0.0391**	0.0293**	0.0156*	0.0283**	0.0299**	0.007

*: P < 0.05

**: P < 0.01.

When an AMOVA was run for 18 populations in three areas in Fukuoka, the percentage variation among the areas was 3.4% (Table 5A; F_ct_ = 0.03442, P < 0.01), while most genetic variation occurred within populations. Post-hoc pairwise comparisons indicated that genetic differentiation was greatest between the container terminal area and the distant area (Table 5A2; F_ct_ = 0.05251, P < 0.01). The genetic differentiation was also significant between the container terminal area and the Chuo terminal area (Table 5A1; F_ct_ = 0.02421, P < 0.01) and between the Chuo terminal area and the distant area (Table 5A3; F_ct_ = 0.03122, P < 0.01). These results suggested that three areas were genetically structured respectively, and the container terminal area was more distinctive than the other two areas. In Kitakyushu, the percentage variation among different areas was small and not significant (Table 5B; F_ct_ = 0.00526, P = 0.1828). Likewise, the percentage variation among the three areas in Nagasaki was small and not significant (Table 5C; F_ct_ = 0.00313, P = 0.13783).

**Table 5:** Results of the AMOVA analysis on the *Ae*. *albopictus* populations in the three cities.

	Source of variation	Sum of squares	Variance components	Percentage variation	statistics	*P*
(A) Fukuoka, three groups (Container terminal, Chuo terminal and Distant areas)						
	Among groups	70.845	0.14843	3.44212	FCT = 0.03442	< 0.01
	Among populations within groups	125.557	0.14791	3.43015	FSC = 0.03552	< 0.01
	Within populations	2147.694	4.01584	93.12772	FST = 0.06872	< 0.01
(A1) Fukuoka, two groups (Container terminal and Chuo terminal areas)						
	Between groups	29.82	0.13542	3.12157	FCT = 0.03122	< 0.01
	Among populations within groups	73.981	0.18295	4.21709	FSC = 0.04353	< 0.01
	Within populations	1180.94	4.01995	92.66133	FST = 0.07339	< 0.01
(A2) Fukuoka, two groups (Container terminal and Distant areas)						
	Between groups	46.372	0.22815	5.25058	FCT = 0.05251	< 0.01
	Among populations within groups	80.633	0.11691	2.69046	FSC = 0.02840	< 0.01
	Within populations	1499.232	4.00012	92.05896	FST = 0.07941	< 0.01
(A3) Fukuoka, two groups (Chuo terminal and Distant areas)						
	Between groups	30.019	0.10375	2.42108	FCT = 0.02421	< 0.01
	Among populations within groups	96.5	0.15234	3.55495	FSC = 0.03643	< 0.01
	Within populations	1615.215	4.0292	94.02396	FST = 0.05976	< 0.01
(B) Kitakyushu, three groups (Tachiura terminal, Hibiki terminal, and Distant areas)						
	Among groups	21.078	0.0231	0.52629	FCT = 0.00526	0.1828
	Among populations within groups	31.723	0.09778	2.22744	FSC = 0.02239	< 0.01
	Within populations	1192.849	4.26899	97.24627	FST = 0.02754	< 0.01
(C) Nagasaki, three groups (Yanagi terminal, Matsugae terminal, and Distant areas)						
	Among groups	21.544	0.01374	0.31306	FCT = 0.00313	0.13783
	Among populations within groups	66.266	0.11393	2.59553	FSC = 0.02604	< 0.01
	Within populations	1701.764	4.26177	97.09141	FST = 0.02909	< 0.01

### Comparison with outside populations using microsatellite analysis

**Fig 2:**
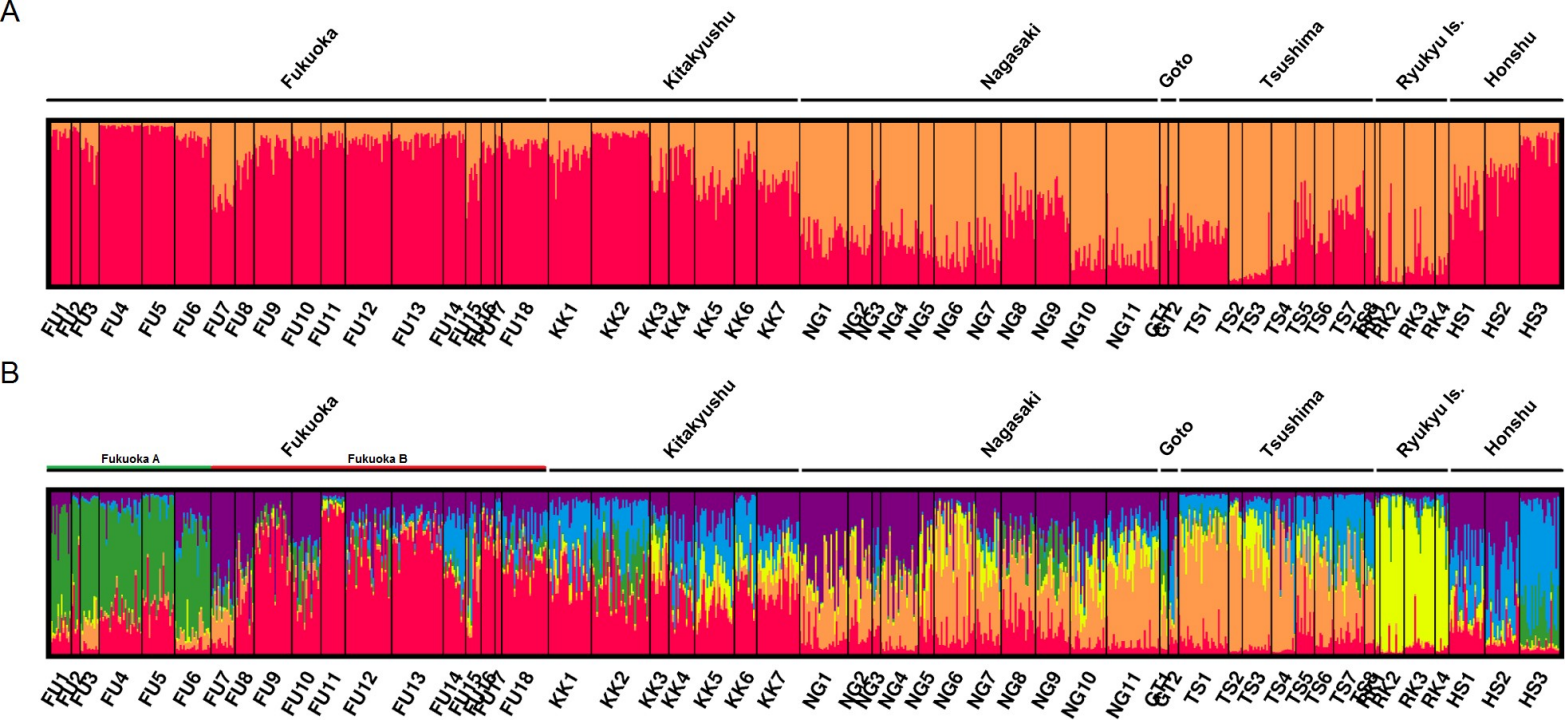
Bayesian membership assignment of *Ae*. *albopictus* individuals from Japan. Only results supported by Evanno’s best K are shown. (A) indicates the cluster assignment with K = 2, red: cluster 1; orange: cluster 2. (B) shows the cluster assignment with K = 6, red: cluster 1; orange: cluster 2; yellow: cluster 3; green: cluster 4; blue: cluster 5; and purple: cluster 6.

**Fig 3:**
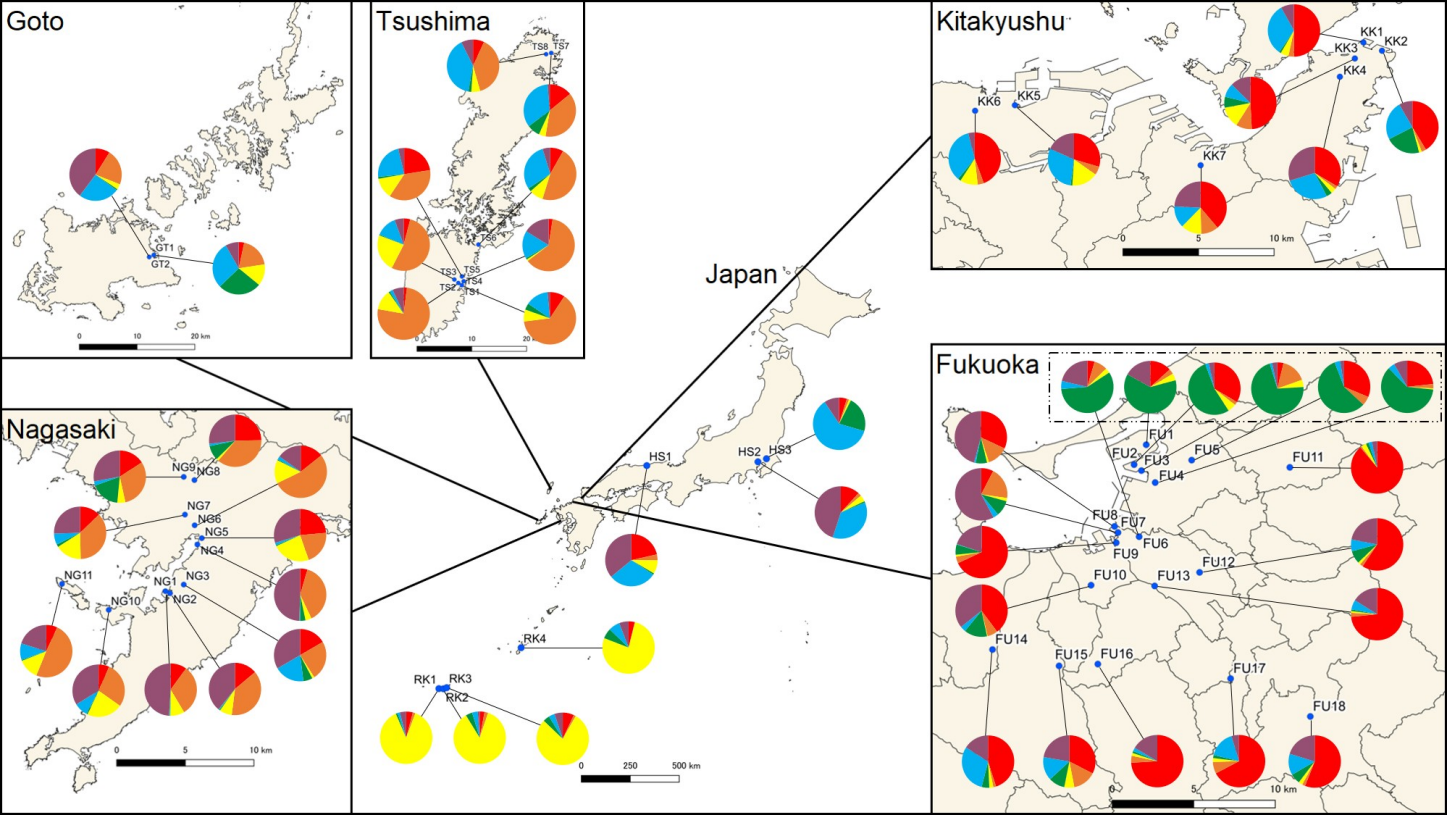
Bayesian membership assignment of *Ae*. *albopictus* populations in Japan with K = 6. Red: cluster 1; orange: cluster 2; yellow: cluster 3; green: cluster 4; blue: cluster 5; and purple: cluster 6. Fukuoka A is enclosed in dashed line. Created by processing Free vector and raster map data @ naturalearthdata.com and National Land Numerical Information (Administrative Area Data) @ Ministry of Land, Infrastructure, Transport and Tourism, Japan (https://nlftp.mlit.go.jp/ksj/gml/datalist/KsjTmplt-N03-v3_0.html).

**Fig 4:**
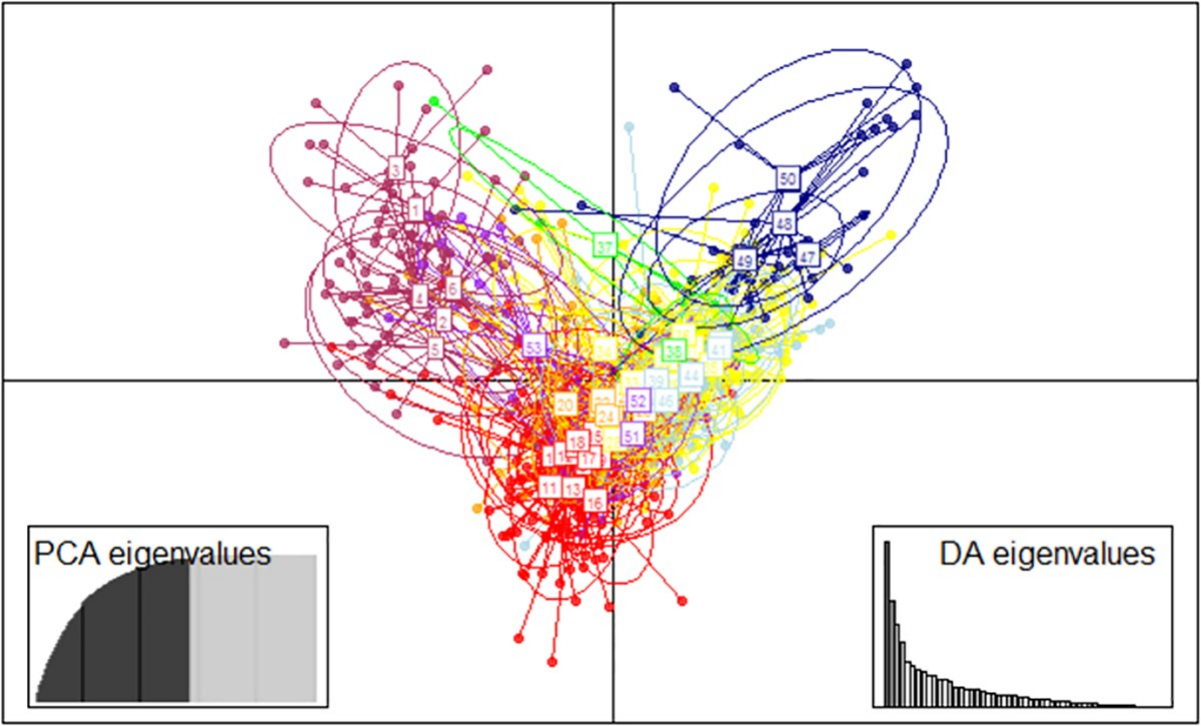
Discriminant analysis of principal components for the *Ae*. *albopictus* populations in Japan. 1–6: Fukuoka A colored by maroon; 7–18: Fukuoka B colored by red; 19–25: KK1-KK7 colored by orange; 26–36: NG1-NG11 colored by yellow; 37–38: GT1-GT2 colored by green; 39–46: TS1-TS8 colored by light blue; 47–50: RK1-RK4 colored by navy blue; and 51–53: HS1-HS3 colored by purple.

K = 2 or 6 was supported by Evanno’s best K in Japanese populations (results including Philippines and Thailand populations shown in S1 and S2 Figs). The cluster analysis with K = 2 recognized two groups: Ryukyu, Goto, Tsushima, and Nagasaki formed one group, and the other group included Fukuoka, Kitakyushu, and Honshu. When K = 6 was applied, the analysis produced six distinct groups. Ryukyu was separated from the other populations; Goto, Tsushima, and Nagasaki formed one group; and Honshu was separated into two groups. Fukuoka populations were separated into two groups: one which forms a distinct group that mainly consisted of Cluster 4 (hereafter, Fukuoka A including FU1 –FU6) and another that consisted mainly of Clusters 1 and 6 (hereafter, Fukuoka B including FU7 –FU18). Composition of the clusters in Fukuoka B was similar to that in the Kitakyushu populations. In detail, Fukuoka A consisted of the container terminal populations, their vicinity populations, and one vicinity population of the Chuo terminal (Figs 2 and 3 and S4 Table). These six populations were within a single continuous area located in the eastern part of Fukuoka city. The discriminant analysis produced three distinct groups: Ryukyu and Fukuoka A formed their own groups, and the remaining populations formed one large group (Fig 4).

**Fig 5:**
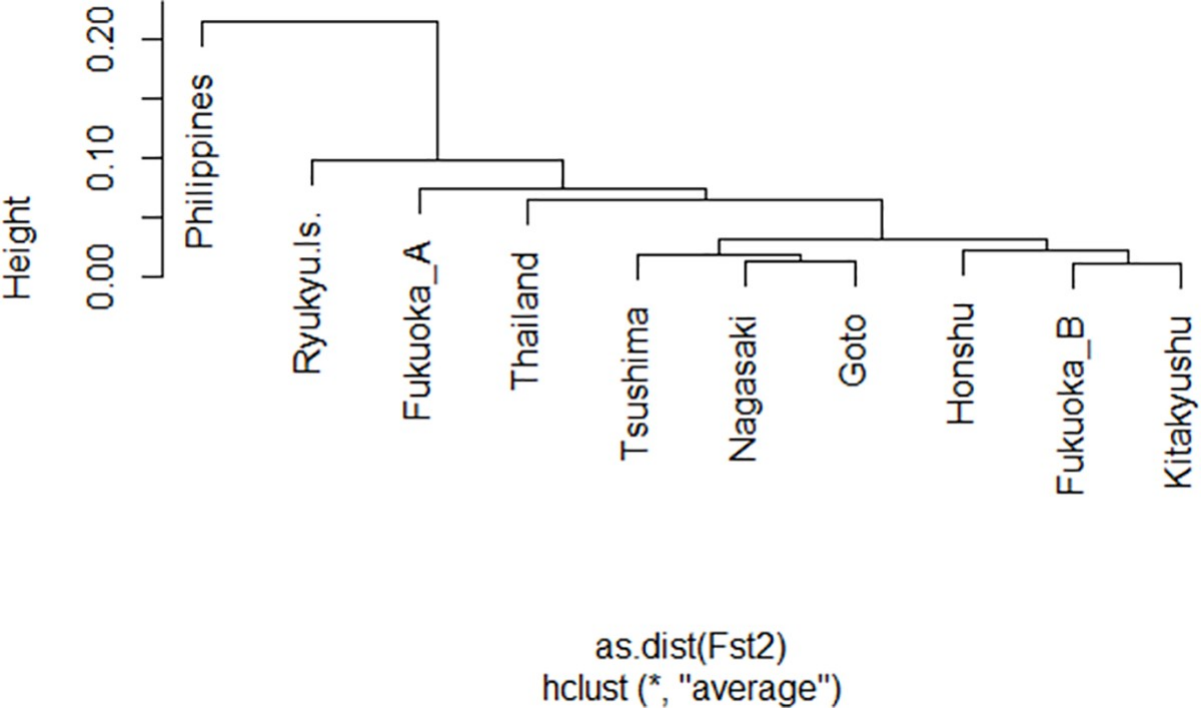
Cluster analysis using F_st_ in ten *Ae*. *albopictus* groups consisting of Kyushu, Honshu, and two tropic populations.

Pairwise F_st_ from each origin was calculated, which included the Philippines and Thailand populations as references and dividing the Fukuoka populations into two groups. The results supported that Fukuoka A had a closer relationship with Fukuoka B (F_st_ = 0.0490, P < 0.01), but Fukuoka B was more genetically similar to other Japanese populations except for the Ryukyu Islands. The F_st_ values with the Philippines, Thailand, and the other groups in Japan were all over 0.05 (P < 0.01) for Fukuoka A populations (Table 6). The F_st_ derived cluster dendrogram by UPGMA showed that Fukuoka A was isolated from other Kyushu and Honshu populations, suggesting that Fukuoka A did not originate from the surrounding populations (Fig 5).

**Table 6:** Pairwise fixation index values (F_st_) between ten *Ae*. *albopictus* groups consisting of Kyushu, Honshu, and two tropic populations.

	Fukuoka_A	Fukuoka_B	Kitakyushu	Nagasaki	Goto	Tsushima	Ryukyu Is.	Honshu	Philippines
Fukuoka_B	0.049**								
Kitakyushu	0.0531**	0.0108**							
Nagasaki	0.0722**	0.0287**	0.0226**						
Goto	0.0873**	0.0436**	0.0264**	0.0136**					
Tsushima	0.0822**	0.0376**	0.0241**	0.019**	0.0176**				
Ryukyu Is.	0.1595**	0.1083**	0.0858**	0.0714**	0.0771**	0.0972**			
Honshu	0.0621**	0.0221**	0.0208**	0.0326**	0.0317**	0.0306**	0.0972**		
Philippines	0.2036**	0.2257**	0.2098**	0.2121**	0.2279**	0.2244**	0.2322**	0.2359**	
Thailand	0.1161**	0.0871**	0.0654**	0.0541**	0.0473**	0.0543**	0.0953**	0.0844**	0.1758**

*: P < 0.05

**: P < 0.01. The values of F_st_ > 0.05 were indicated in bold.

### Comparison with outside populations using CO1 analysis

**Fig 6:**
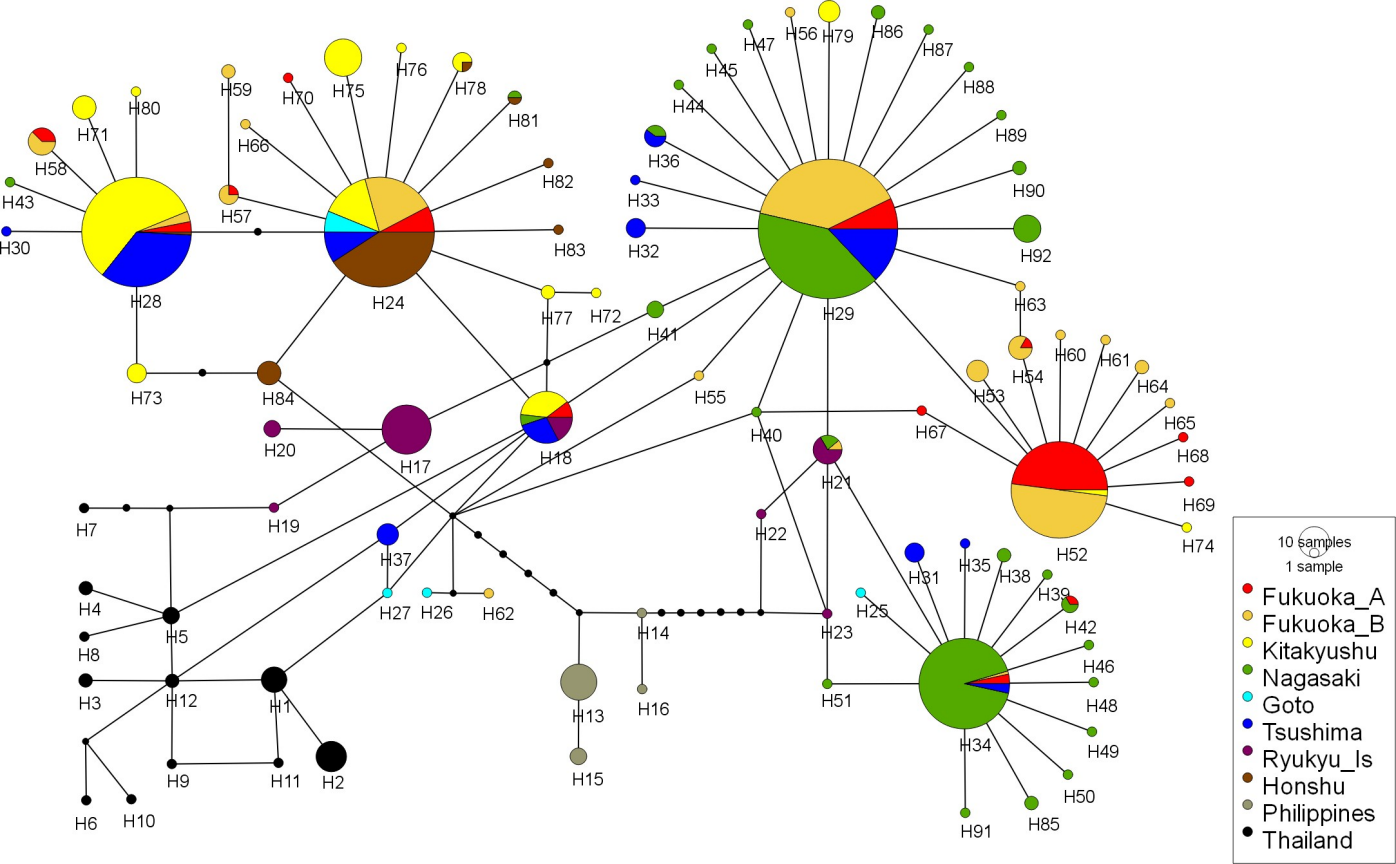
CO1 genetic relationships among ten *Aedes albopictus* groups.

CO1 sequences were 1,326 bp length. In total, 92 haplotypes were recorded and registered within the DNA Data Bank of Japan (DDBJ; accession number: LC591859-LC591942, LC597549-LC597556). Thailand (H1-12) and Philippines (H13-16) did not share haplotypes with each other or with the Japanese populations (H17-92; Fig 6). Among the Japanese populations, the predominant haplotypes and haplotype compositions varied (S5 Table and S3 Fig). Three major haplotypes (H24, H28, and H29) were widely found among Japanese populations (Fig 6). H52 was found almost exclusively among the Fukuoka populations and two Kitakyushu populations (Fig 6). This haplotype was most common in Fukuoka A (48/93), whereas it was less common in Fukuoka B (50/196) (χ^2^ = 18.029, df. = 1, P < 0.01). The most common haplotype in Fukuoka B (82/196) was H29, and it was less common in Fukuoka A (15/93) (χ^2^ = 17.558, df. = 1, P < 0.01). However, unlike the results of the microsatellite analysis, the F_st_ analysis based on haplotype composition did not clearly divide the Fukuoka populations (S6A–S6C Tables).

**Fig 7:**
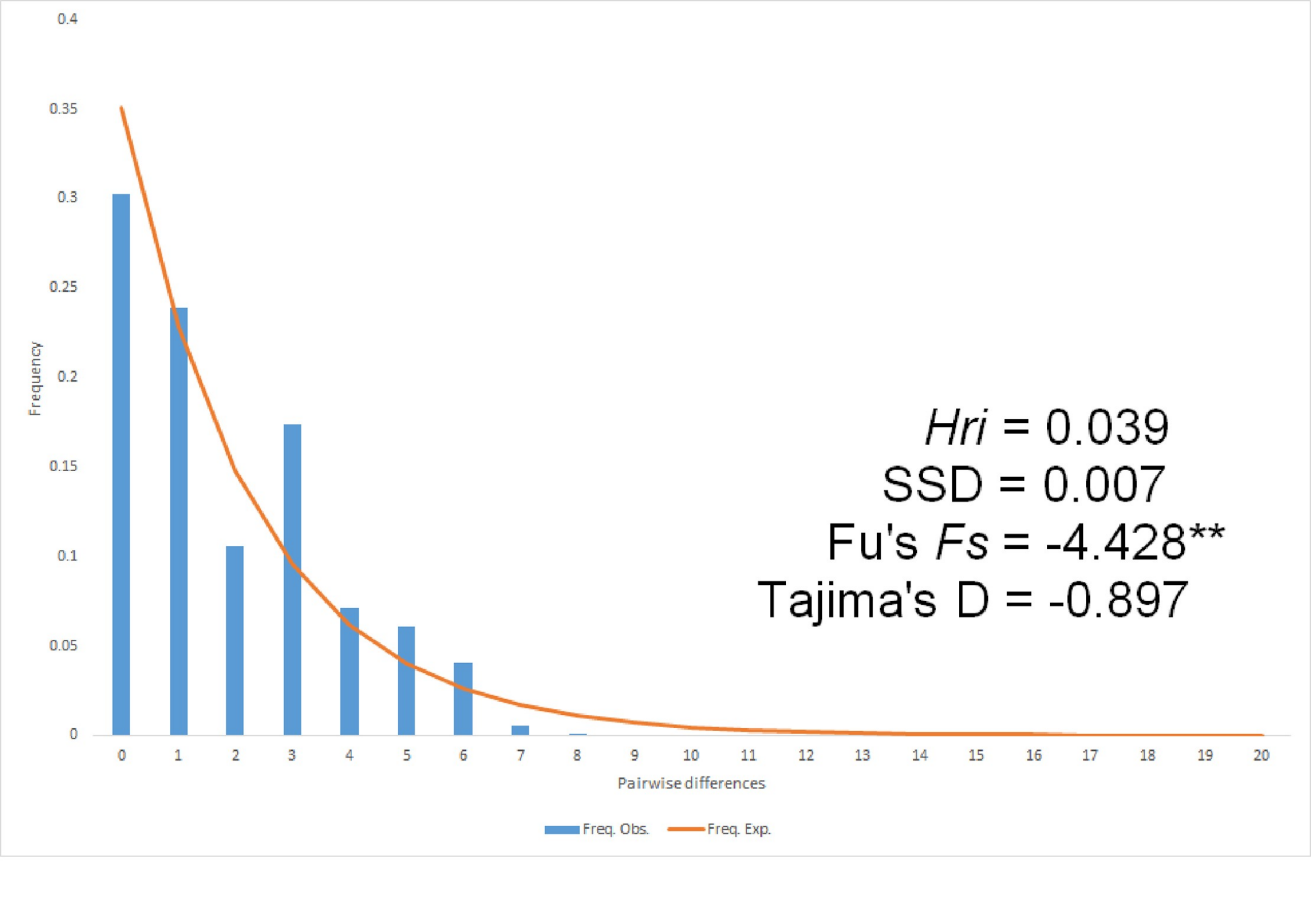
Observed and expected mismatch distribution of the Fukuoka A group.

To infer the history of Fukuoka A, deviations from selective neutrality and mismatch distribution were evaluated. The analysis with Fu’s *Fs* suggested a demographic expansion of Fukuoka A (P < 0.01), but the analysis with Tajima’s D did not suggest the expansion (P > 0.05) (Fig 7). The bimodal mismatch distribution had nonsignificant SSD and *Hri* values, which rejected a sudden population expansion and suggested a stepwise growth of the population; thus, the group has likely been in Fukuoka for a long time (Fig 7).

### Inference of putative sources of the possible introduced group

**Fig 8:**
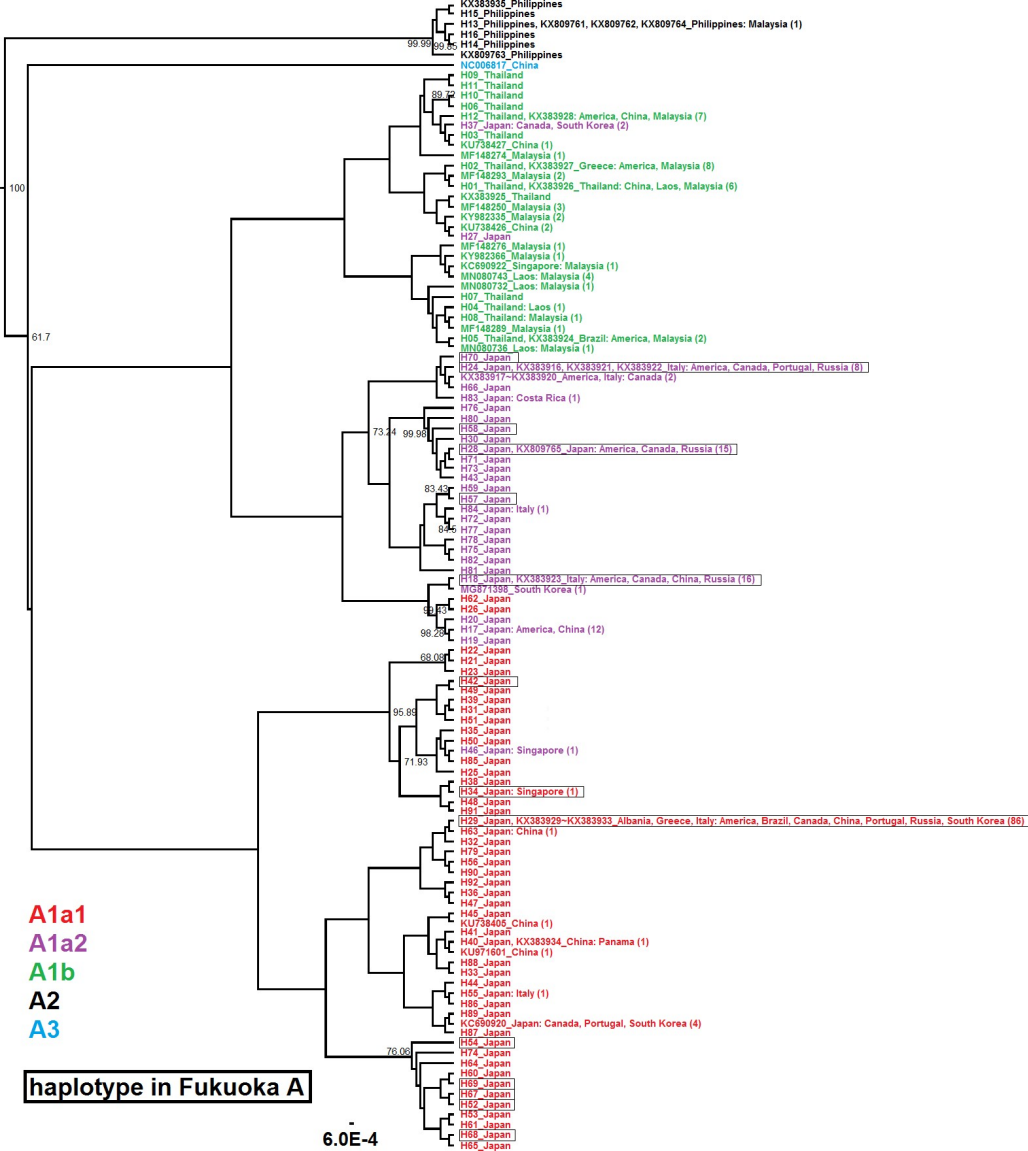
Phylogenetic tree of *Ae*. *albopictus* (total 108 taxa). Taxa are labeled with accession numbers and countries or haplotype name (H1-H84) and countries, and colored by haplogroups as defined by Battaglia et al. [60]. Areas which had identical sequences are listed after a colon symbol. Figures in parentheses show the number of identical sequences found in the 524 sequences. Support values above the branches indicate posterior probabilities (>50) of Bayesian Inference with MCMC. Haplotypes found in Fukuoka A are highlighted (enclosed in boxes).

The phylogenetic tree showed that the most common haplotype H52 in Fukuoka A belonged to haplogroup A1a2 which occurs mainly in the temperate regions of East Asia and some colonized areas such as Europe and North America. All other haplotypes found in Fukuoka A belonged to A1a1 or A1a2, which also occur among the populations in the temperate regions of East Asia and the colonized areas. None of the Japanese populations in the present study harbored haplotypes belonging to A1b, A2, or A3, which occur mainly in the tropical populations of this species (Fig 8).

## Discussion

This study identified one genetically distinct group of *Ae*. *albopictus* (Fukuoka A) in the vicinity of the international terminals in Fukuoka. The five populations near the container terminal (FU1-5) showed distinctness in F_st_ and F_ct_ values based on microsatellite analysis. Another population near Chuo terminal (FU6) was similar to these five populations in cluster and discriminant analyses. As Fukuoka A and B are separated by less than 3 km without physical barriers such as a hill, it is plausible that Fukuoka A originated from outside of the study collection areas rather than being derived from Fukuoka B parapatrically. The establishment of Fukuoka A appears to have been too recent to mix genes homogeneously with the local population. However, the results of Fu’s *Fs* tests, Tajima’s D, and the atypical shape of mismatched distribution suggest that it was not a recent introduction event. The genetic feature of the introduced population endured rather than vanished, possibly because the population size of the founder was not small, the frequency of introduction was not low, or the introduced individuals could reproduce in suitable breeding sites without conspecific species.

It is of interest to determine the origin of Fukuoka A. Our results showed that Fukuoka A was genetically different from the other Japanese and tropical populations. Other Japanese populations except for Ryukyu Islands showed small genetic differences (F_st_ < 0.05, Table 6 and Fig 5). Cluster dendrogram using F_st_ showed that Fukuoka B was similar to Kitakyushu, a neighbor city (45 km apart) of Fukuoka. The genetic structure of the island populations (Goto and Tsushima) was similar to Nagasaki populations, possibly the result of historical human-mediated transportations. These results indicate a general intraregional-associated genetic pattern in Japanese *Ae*. *albopictus* such that close populations showed genetic similarity. However, the genetic profile of Fukuoka A did not fit that intraregional-associated pattern; and was genetically quite dissimilar from other Japanese populations regardless of geographical distance, as tested by Bayesian clustering, discriminant analysis, and F_st_. Fukuoka B is the most genetically similar population to Fukuoka A, but this is presumed to be caused by some limited degree of gene flow between the two groups. Considering that Fukuoka A was distributed near the international terminal, it is likely that the origin of Fukuoka A was outside of Japan. We cannot deny the possibility of the introduction from other areas of Japan such as northern Honshu, Shikoku, and Ogasawara islands. However, it is difficult to propose that Fukuoka A populations were introduced from those areas; considering that the northern Honshu population has expanded recently from the Honshu population [62], Shikoku is an island next to Kyushu, and there is no direct connection by ship between the Ogasawara Islands and Fukuoka. Further research to other areas will help to understand the genetic structure of *Ae*. *albopictus* in Japan and might yield more signs of possible introduction.

The proportions of dominant haplotypes of CO1 in Fukuoka A and B were significantly different, and support the outside-origin results generated by microsatellite analysis. The phylogenetic tree showed that the most common haplotype (H52) and all other haplotypes of Fukuoka A were within clades of roughly temperate areas excepting several tropical areas containing colonized regions. Moreover, according to the definition of a haplogroup per Battaglia et al. [60], we assigned our haplotypes into specific haplogroups based on diagnostic mutant sites within the CO1 fragment. Haplotypes in Japan included H52 and the derived haplotypes all belonged to the A1a1 and A1a2 haplogroups (Fig 8). Those two haplogroups were mainly distributed in native areas of China, Japan, and South Korea, as well as European countries and the Americas in non-native areas, which was considered as a temperate origin and had spread worldwide [60]. Philippine haplotypes all belonged to the A2 haplogroup, and Thai haplotypes all belonged to the A1b haplogroup (Fig 8). A1b and A2 haplogroups were believed to originate from tropical areas [60]. Therefore, those findings further support a temperate origin of Fukuoka A. The tropical origin is additionally unlikely because it would be difficult for tropical populations to overwinter in the Japanese climate. H52 is the most abundant haplotype in Fukuoka A, and its origin could not be inferred due to the absence of identical sequences within GenBank. As H52 had just one step mutation changed from H29, which is predominant in Fukuoka B, it is possible that H52 was derived from H29 in Fukuoka. However, it is also possible that the origin of H52 is outside Japan because the identical haplotype of H29 was frequently found in China, South Korea, and several western countries invaded by *Ae*. *albopictus* (Fig 8). Also noteworthy is that several Japanese haplotypes (H26, H27, H37, H46, and H62) were placed into seemingly inappropriate clades (Fig 8), and the underlying mechanism requires exploration. H26, H27, and H62 were private haplotypes in Goto and Fukuoka (S5 Table), and therefore random mutation may be an explainable factor. However, H37 was found in Tsushima, South Korea, and Canada. Shipping connections exist between Tsushima and South Korea, and H37 might be imported from this region. H46 was detected in Nagasaki as well as in Singapore, which may hint at gene flow of *Ae*. *albopictus* via human-mediated transportations between these sites.

A genetically distinct population were found in Fukuoka, but not in Kitakyushu or Nagasaki. This suggests that large introduction may be required to establish an exotic population. Frequent introduction may be related to transportation type and the nature and magnitude of cargo traffic might be better than passenger ships for facilitating international movement of *Ae*. *albopictus*. This notion is explained by the amount of container cargo handled by the Fukuoka container terminals. The Fukuoka terminals are located next to each other, and their combined cargo traffic is 6 to 7 times greater than each Kitakyushu terminal. The amount of cargo handled by the Nagasaki terminal was relatively small. Although the Fukuoka Chuo terminal and the Nagasaki terminals host the first and second largest numbers of cruise ships in Japan, respectively, the results suggest that mosquitoes are less likely to be introduced by cruise ships. Closed freight containers may provide more protected space for trapped mosquitoes, while cruise ships have better hygiene and open environment to the sea, which may reduce the chance of importing mosquitoes. The high volume of cargo shipping in Fukuoka might enhance the opportunity for introduction of exotic *Ae*. *albopictus*. Furthermore, if a population is introduced from a similar temperate environment, it might survive by repeated introductions in the seaport areas of Japan.

Most containers handled by the Fukuoka terminals originated from Asian countries, mainly China and South Korea. Those introduced individuals with greater vector competence/insecticide resistance should be given more attention. Specifically, there were several reports of *kdr* of *Ae*. *albopictus* in China [27,28], and this should alert the possibility of introduction of insecticide resistant populations.

### Limitation

The small sample sizes at some sites might have limited precise estimations of their F_st_ values. The simulation by Hale et al. suggested that 25 to 30 individuals are needed to estimate allele frequencies with 5–9 microsatellite markers [63]. Because our study used 13 microsatellite markers, we think that the sample size limitation does not give serious issue for our conclusion. As the CO1 technique works better with data from a wider area, more samples from various sites within and outside of Kyushu, especially from China and South Korea, are needed to maximize the potential of the technique for more precisely inferring the origin of Fukuoka A. Goubert et al [32] proposed strengthening the worldwide collaborations among scientists to find better universal and informative markers for *Ae*. *albopictus*, which will help to delineate a better picture of the worldwide movements of this mosquito species.

## Conclusion

The present study found a sign of *Ae*. *albopictus* introduction through maritime freight container transportation. The populations found in the international container terminals in Fukuoka likely originated from a temperate region outside of Japan. However, the populations in the international terminals in Kitakyushu and Nagasaki were not genetically distinct from the other populations. As vector competence varies among different populations [21], and the knock down resistance gene has been reported from some populations outside Japan [24–29], the chance of introducing greater vector competence/insecticide resistant mosquitoes into Japan should be considered. This is the first study to describe the spatial population structure of *Ae*. *albopictus* in Japan using molecular techniques. Further study will give us more detailed information of population structure and ongoing introduction of this mosquito species in Japan.

## Supporting information

S1 Table List and characteristics of the microsatellite primers used in the study. (XLSX)

S2 Table Microsatellite scores of each locus in this study. (XLSX)

S3 Table Estimates of genetic diversity of *Ae*. *albopictus* populations using microsatellite markers. Displayed are the mean values of each population and the standard error (SE). N: mean populations size; N_a_: mean number of alleles; N_e_: number of effective alleles; H_o_: observed heterozygosity; H_e_: expected heterozygosity; uH_e_: unbiased expected heterozygosity; F: fixation index.
(XLSX)

S4 Table Membership coefficient of each population when best K = 6. (XLSX)

S5 Table CO1 haplotype distribution in distinct geographical populations. (XLSX)

S6 Table (A) Pairwise fixation index (F_st_) of CO1 among eighteen *Ae*. *albopictus* populations in Fukuoka, (B) Pairwise fixation index (F_st_) of CO1 among seven *Ae*. *albopictus* populations in Kitakyushu, and (C) Pairwise fixation index (F_st_) of CO1 among eleven *Ae*. *albopictus* populations in Nagasaki. (XLSX)

S1 Fig Bayesian membership assignment of *Ae*. *albopictus* individuals from Japan. Only results supported by Evanno’s best K are shown. (A) indicates the cluster assignment with K = 2, red: cluster 1; and orange: cluster 2. (B) shows the cluster assignment with K = 8, red: cluster 1; orange: cluster 2; yellow: cluster 3; green: cluster 4; blue: cluster 5; purple: cluster 6; brown: cluster 7; and grey: cluster 8.
(TIF)

S2 Fig Discriminant analysis of principal components of *Ae*. *albopictus*. 1–18: FU1-FU18 colored by red; 19–25: KK1-KK7 colored by orange; 26–36: NG1-NG11 colored by yellow; 37–38: GT1-GT2 colored by green; 39–46: TS1-TS8 colored by light blue; 47–50: RK1-RK4 colored by navy blue; 51–53: HS1-HS3 colored by purple; 54: PHL colored by brown; and 55: THA colored by black.
(TIF)

S3 Fig Haplotypes distribution of *Ae*. *albopictus* in Japan. Created by processing Free vector and raster map data @ naturalearthdata.com and National Land Numerical Information (Administrative Area Data) @ Ministry of Land, Infrastructure, Transport and Tourism, Japan (https://nlftp.mlit.go.jp/ksj/gml/datalist/KsjTmplt-N03-v3_0.html).
(TIF)
