# Supplementary figures and images for: Searching for a sign of exotic Aedes albopictus (Culicidae) introduction in major international seaports on Kyushu Island, Japan
Source: PLoS Negl Trop Dis. 2021 Oct 6;15(10):e0009827. doi: 10.1371/journal.pntd.0009827 (PMC8523054; doi:10.1371/journal.pntd.0009827)

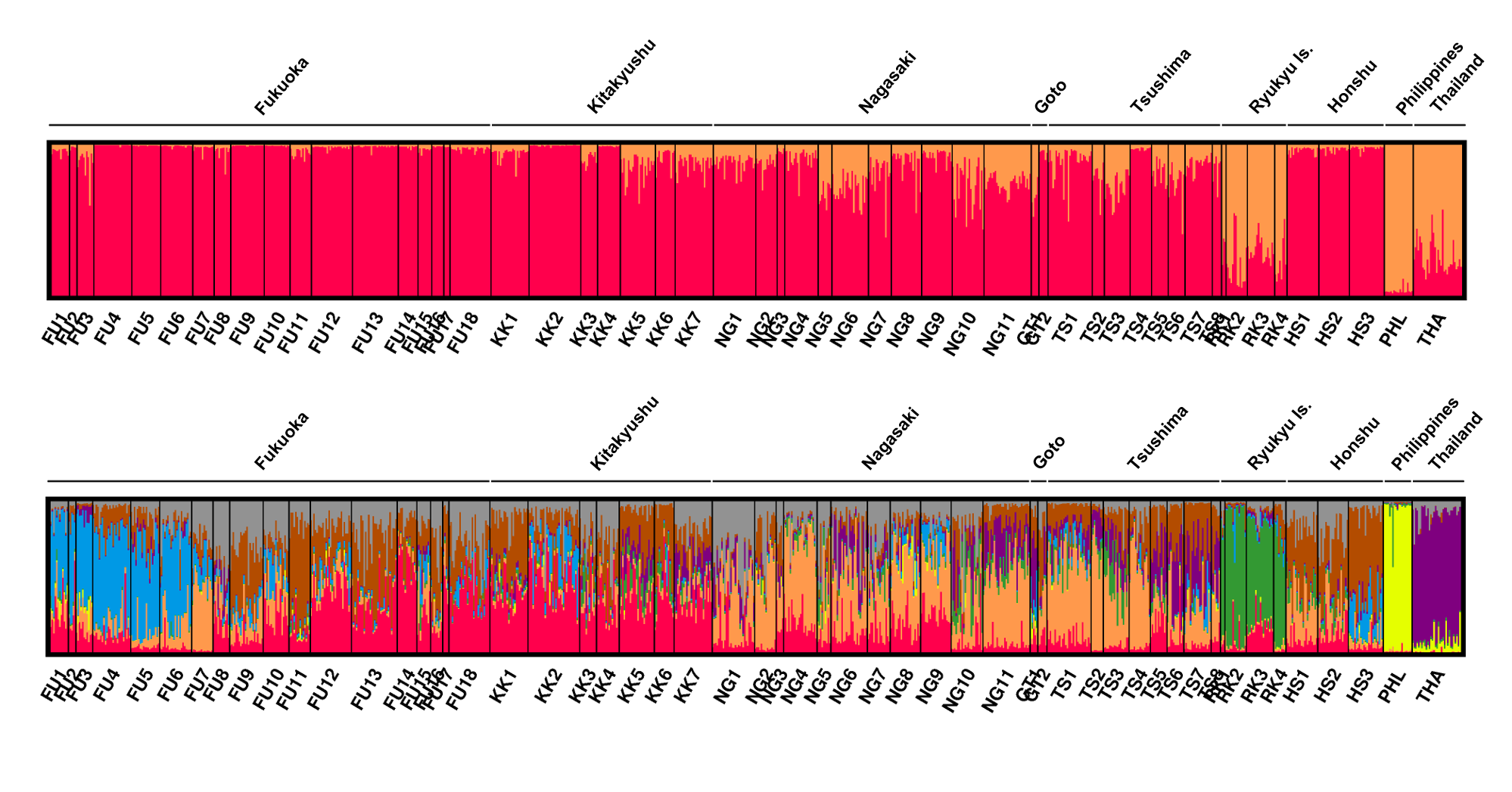

Supplement: S1 Fig — Only results supported by Evanno’s best K are shown. (A) indicates the cluster assignment with K = 2, red: cluster 1; and orange: cluster 2. (B) shows the cluster assignment with K = 8, red: cluster 1; orange: cluster 2; yellow: cluster 3; green: cluster 4; blue: cluster 5; purple: cluster 6; brown: cluster 7; and grey: cluster 8. (TIF) [file pntd.0009827.s007.tif]

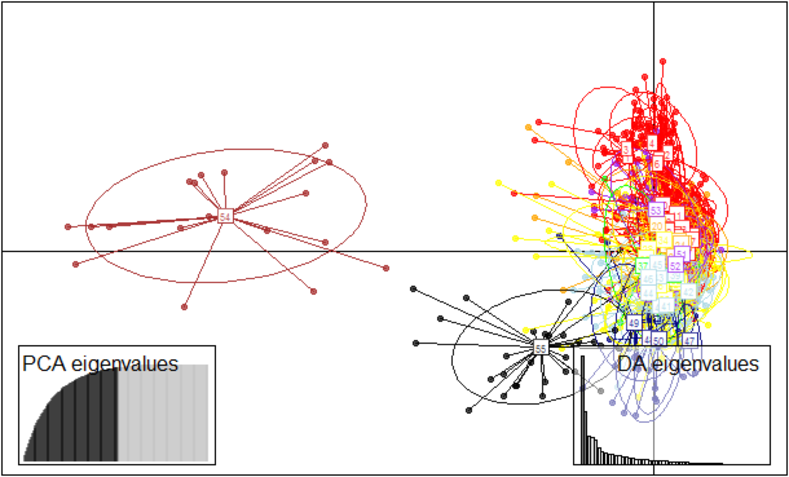

Supplement: S2 Fig — 1–18: FU1-FU18 colored by red; 19–25: KK1-KK7 colored by orange; 26–36: NG1-NG11 colored by yellow; 37–38: GT1-GT2 colored by green; 39–46: TS1-TS8 colored by light blue; 47–50: RK1-RK4 colored by navy blue; 51–53: HS1-HS3 colored by purple; 54: PHL colored by brown; and 55: THA colored by black. (TIF) [file pntd.0009827.s008.tif]

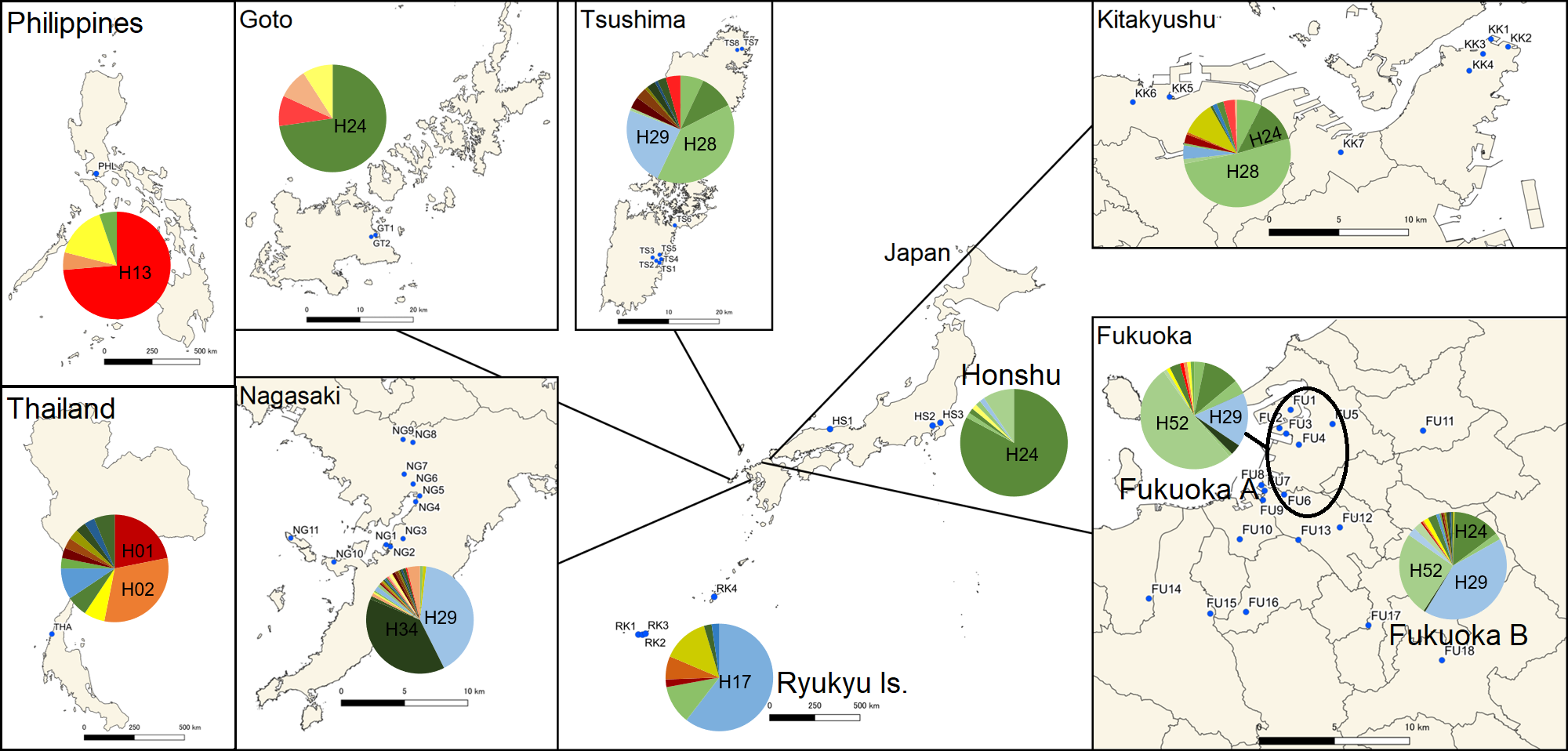

Supplement: S3 Fig — Created by processing Free vector and raster map data @ naturalearthdata.com and National Land Numerical Information (Administrative Area Data) @ Ministry of Land, Infrastructure, Transport and Tourism, Japan (https://nlftp.mlit.go.jp/ksj/gml/datalist/KsjTmplt-N03-v3_0.html). (TIF) [file pntd.0009827.s009.tif]
